# Whole genome sequencing approach for the detection of rare protein-altering variants associated with familial male infertility

**DOI:** 10.1186/s43042-025-00828-y

**Published:** 2025-12-31

**Authors:** Muhammad Ajmal, Muhammad Riaz Khan, Arvand Akbari, Helen Castillo-Madeen, Jasvinder S. Ahuja, Taqweem Ul Haq, Donald F. Conrad, Aftab Ali Shah

**Affiliations:** 1 Department of Biotechnology, Faculty of Biological Sciences, University of Malakand, Chakdara, Khyber Pakhtunkhwa, Pakistan; 2 Center for Embryonic Cell & Gene Therapy, Oregon Health & Science University, Portland, OR, USA; 3 Division of Genetics, Oregon National Primate Research Center, Beaverton, OR, USA; 4 Department of Molecular and Medical Genetics, Oregon Health & Science University, Portland, OR, USA

**Keywords:** Familial infertility, Genotyping, Next-generation sequencing, Whole genome sequencing

## Abstract

**Background:**

Whole-genome sequencing (WGS) is an efficient, accurate, and high-throughput technique used for the discovery of exonic as well as non-exonic variants causing different genetic anomalies, improving clinical management for the disease. This study was designed to evaluate pathogenic genetic variants related to male infertility in male infertile families in remote rural areas of Pakistan.

**Methods:**

For the five families recruited, WGS was conducted on 14 patients and 6healthy controls. WGS was performed using the Illumina TruSeq DNA Nano Library Prep workflow protocol. DNA was assessed with TapeStation and sequenced using the TruSeq kit (Illumina). Bioinformatics tools were then used to identify potential causative variants, including indels and single-nucleotide variants.

**Results:**

In family 1, five homozygous nonsynonymous SNVs were identified in *FMR1*, *PHF8*, *TOPBP1*, and *DAB2IP*. These X-linked or recessive variants cause amino acid changes that affect key cellular pathways. In family 10, homozygous nonsynonymous SNVs were detected in *FOS*, *MIR452*, *PDHA1*, *and SERPINB1*, implicating roles in structural, transcriptional, metabolic, and synaptic processes. In family 11, a frameshift deletion in *PRDM9* and nonsynonymous SNVs in *KIAA1210*, suggesting possible disruption of gene regulation and protein stability. In family 13, multiple nonsynonymous variants were identified in *SPATA20*, *and G6PD*,, with implications in reproductive function, angiogenesis, enzymatic activity, and neuromuscular disorders. SNV Variant was detected in *SLC12A4* in Family 15.

**Conclusion:**

The study contributes to a systematic understanding of the problem and provides a basis for identifying causal variations in idiopathic cases of male infertility, setting targets for future therapeutic development.

## Introduction

In human life, reproduction is one of the central events linked with the pivotal aim of delight, totality, and family consolidation. A significant measure of effective reproduction in humans is fertility outcome [1]. Related to the human civilization, infertility is a key health problem that extremely impacts the psychological, social, and economic well-being of those experiencing it and their families as well [2]. Failure of vibrant couple to achieve pregnancy after twelve months of unprotected sexual intercourse is termed as infertility according to the World Health Organization (WHO) [3] and is documented as a disorder of the reproductive system by the International Committee for Monitoring Assisted Reproductive Technology (ICMART), as well as by the World Health Organization (WHO). Across the globe, almost 50 million couples are reported as victims of infertility. Infertility prevalence is very high, worldwide, affecting 8–12% of couples of reproductive age.

Pakistan, being the fifth-most populous country in the world, has a population of more than 249 million having trend of consanguineous marriages between first or second cousins due to local norms and traditions. Conservative estimates report that about 70% of marriages in Pakistan are consanguineous [4]. These marriages enhance the probability of homozygous variants in the offspring as compared to marriages between non-relatives. Offspring resulting from consanguineous marriages show about 10% higher incidence of inbred deformities, instigating multiple disorders. In Pakistan, the prevalence of primary infertility accounts for 4% of the total 22% reported previously [5]. However, this frequency may be higher in the local population, equating the total population and global prevalence (50 million couples) of infertility [6]. Both male and female factors are attributed to infertility, adversely distressing reproductive ability (WHO). Both partners equally contribute to the etiology of infertility [7, 8]. In seeking male infertility, half of the infertility cases are idiopathic.

Male infertility is also subject to several environmental factors like stress, lifestyle, eating habits, alcohol abuse, and smoking [9]. Previous studies have identified several candidate genes for male infertility [10]. The complete and accurate genetic information required for fertility maintenance is not yet determined. Recent genomic screening and homozygosity mapping were used for the detection of variants in inherited retinal disease in the same study population [11]. Functional studies will be required to validate the role of these detected variants.

A complex network of genes regulates all biological functions in a coordinated way [12]. Spermatogenesis and spermiogenesis are also tackled in this way; more than two thousand genes are reported to be entangled in the spermatogenesis process, and a huge number of genes are assumed to be required for its maintenance [13]. Sperm production from primordial germ cells is called spermatogenesis. Abnormal spermatogenesis results due to irregularity in the complex multi-step process of spermatogenesis [14]. Certain genes present on unrelated chromosomes can regulate gene expression on remote loci. Whole genome sequencing aids in discovering regulatory elements present on unrelated chromosomes that yet control the expression of genes involved in causing infertility [4]. Certain disrupted genes on autosomes can be enriched for undescribed human “knockouts” and are yet to be linked to the infertility phenotype. *Tuba3a/3b* knock-out mice completely lost germ cells at the early postnatal stages [15]. The current WGS study identifies a candidate variant (different genes) in related men suffering from infertility. This claim is further substantiated by an analysis of data from single-cell expression data from HISTA (https://conradlab.shinyapps.io/HISTA/). An online database of human testicular gene expression [16].

## Materials and methods

### Inclusion and exclusion criteria, recruitment of the families, and clinical characteristics

The Departmental Bioethical Committee (DBC), University of Malakand, has reviewed and approved the current study vide notification No. UoM/BT/BC/2019/20 dated July 4, 2019. Furthermore, the DBC was endorsed by the Advanced Studies and Research Board (ASRB) of the University of Malakand, Pakistan (Ref. No. UOM/Admin/2022/112 dated February 02, 2022). Written informed consent was taken from all patients and healthy controls of selected families according to the ethical standard outlined in the Declaration of Helsinki [17]. Probands from each family were first identified through preclinical data collected in the local community of Malakand Division, Khyber Pakhtunkhwa, Pakistan. Family members with a history of infertility were examined by an Andrologist and were recruited as shown in Fig. 1. The semen analysis was conducted in a healthcare laboratory as per World Health Organization (WHO) criteria. The semen examination was performed immediately after the liquefaction of semen and no longer than 60 min after ejaculation. The semen was examined microscopically, and the reports were generated accordingly. Detailed information about the clinical data of patients diagnosed with Azoospermia included in this study is shown in Table 1; Fig. 2. Additionally, detailed family histories were obtained to exclude any potential inflammatory, infectious, or accidental risk factors before proceeding with genetic analyses.

### Blood sample collection and genomic DNA extraction

A total of 3–5 mL whole blood samples were collected from patients and healthy controls aseptically in an EDTA tube. Genomic DNA was extracted through the Phenol/Chloroform method [18], and the extracted DNA was confirmed through gel electrophoresis (Clever Scientific Ltd, UK) as reported previously [19].

### Library preparation and sequencing for NGS

The Illumina TruSeq DNA Nano Library Prep workflow protocol was used for WGS. After checking the qualitiy of the extracted DNA, the samples were subjected to library preparation using the TruSeq Nano DNA Library Prep Kit (Illumina). Sonicator was used for the fragmentation of DNA using the S220 sonicator (Covaris). The fragmented DNA was subjected to end-repair and size selection (approximately 200–700 bp), followed by 3′-end adenylation and ligation of indexed sequencing adapters. The adapter-ligated fragments were subsequently amplified through a limited number of PCR cycles. The quality and quantity of the resulting libraries were then assessed before sequencing by the Bioanalyzer TapStation (Agilent). The libraries were normalized, pooled, and sequenced using the NovaSeq 6000 platform. Raw base call files were then converted into FASTQ format using bcl2fastq (Illumina).

### GATK pipelines for NGS data analysis

Raw FASTQ files were uploaded to the cloud environment, where they were processed into genomic Variant Call Format (gVCF) files. Briefly, this workflow follows steps analogous to the pipeline, encompassing read mapping and alignment, sorting and duplicate marking, variant calling, and variant quality score recalibration (VQSR). Single-family Variant Call Format (VCF) files containing SNVs and insertions/deletions (indels) were subsequently generated through joint genotyping. Furthermore, CNVs were detected using smoove (https://github.com/brentp/smoove).

#### Variant filtering and annotation

As previously reported [4], whole-genome sequencing and bioinformatics analysis were performed according to the approved protocol [19]. The NGS data were filtered for rare recessive variants with a minor allele frequency (MAF) of < 0.01, CADD Score of > 20, Genotype; Homo, Disease Model; REC-Hom, Variant Location; Exonic, Gene Expression; Expressed in Testis and Literature; PubMed. The target regions were amplified in the candidate genes using conventional PCR [20]. Investigating the expression of candidate genes using the human infertility single-cell testis atlas (HISTA). It is an interactive web tool for a better understanding of human spermatogenesis through scRNA-Seq analysis. It represents the expression level and location of candidate biomarkers in different cells during spermatogenesis. With 26,093 high-quality cells taken from testis biopsies, the human infertility single-cell testis atlas (HISTA) web portal (2022 data release) (https://conradlab.shinyapps.io/HISTA/) [21] is an interactive web tool for understanding human spermatogenesis through scRNA-Seq analysis. HISTA also comprises machine-learning-derived gene modules that capture the entire transcriptional perspective of the testis tissue. We show how the use of these gene modules/components can simplify the highly complex data in HISTA, assist with the interpretation of genes with unknown functions, and allow for hypothesis testing. In the present study, the expression of different genes was investigated using HISTA as reported previously [19].

## Results

### Variant filtering

From six collected families (3−5 individuals per family), twenty-three individuals, including either one or two healthy controls within each family, were processed to WGS. Variants segregating in the family, leading to altered protein structure, were identified as candidate variants. A total of four, six, three, five, two, and two candidate genes were reported for families no. 1, 10, 11, 13, and 15, respectively. Six filters were stringently appliedto select the best candidates. These filters include the CADD Score.

(> 20), Genotype (Homo), Disease Model (REC-Hom), Variant Location (Exonic), Gene Expression (Expressed in Testis) and Literature (PubMed). The CADD score, Disease Model, rs No, and the American College of Medical Genetics and Genomics (ACMG) Guidelines for Variant classification as shown in Table 2.

### Family no.1

WGS was performed on the DNA of four individuals, including 1–1, 1–2, 1–3, and 1–4. A total of 2319, 2111, 2359, and 2264 variants were obtained, respectively.

#### Identification of nonsynonymous SNV in FMR1, PHF8, TOPBP1 and DAB2IP

Our study identified a nonsynonymous single-nucleotide variant (SNV) (rs587780338) in the *Fragile X* Messenger Ribonucleoprotein 1 (*FMR1)* gene, located on chromosome X (Xq27.3, Genomic coordinates (GRCh38): X:147,909,431 − 147,911,817) at position 147,948,696. This gene comprises 17 exons. This variant causes different amino acid substitutions in several *FMR1* transcripts, such as p.C563Y, p.C584Y, p.A494T, and p.C538Y. The inheritance pattern is X-linked homozygous with a CADD score of 22.7, suggesting a potentially deleterious effect. This variant may contribute to phenotypic manifestations associated with fragile X syndrome or related neurodevelopmental disorders. The normal and mutated protein structure is shown in Fig. 3.

Our study revealed a nonsynonymous SNV in the *PHF8* gene, located on chromosome X (Xp11.22, Genomic coordinates (GRCh38): X:53,936,680 − 54,048,936) at position 53,985,021. The total exons in the *OHF8* gene is 23. This variant leads to multiple transcript-specific amino acid substitutions, such as p.P678L, p.P815L, p.P779L, and p.P762L, in several *PHF8* transcripts (ENST00000338946.10, ENST00000357988.9, ENST00000338154.11, and ENST00000322659.12). This variant is a X-linked homozygous. *PHF8* plays a pivotal role in chromatin remodeling and is linked with X-linked intellectual disability syndromes, such as Siderius type X-linked mental retardation.

We identified a homozygous nonsynonymous SNV (rs1353662227 C > G) in the *TOPBP1* gene located on chromosome 13 (3q22.1, Genomic coordinates (GRCh38): 3:133,600,238 − 133,661,941) at position 133,652,615. This variant leads to an amino acid substitution from aspartic acid to histidine at position 313. The transcript is ENST00000260810.10. This variant is in a homozygous state and follows a recessive mode of inheritance. This variant may affect the DNA damage response pathway regulated by *TOPBP1*, a critical factor in genome stability and cell cycle control. Given its functional annotation and predicted pathogenicity.

A homozygous nonsynonymous SNV (rs183735294 C > T) in the *DAB2 interacting protein (DAB2IP)* gene was identified, located on chromosome 9 (9q33.2, Genomic coordinates (GRCh38): 9:121,567,074–121,785,530) at position 121,773,239, and comprising 18 exonsand occurs in exonic regions of several transcripts, such as ENST00000309989.1, ENST00000259371.6, ENST00000648444.1, and ENST00000408936.7, resulting in the amino acid substitutions p.T780M, p.T876M, p.T843M, and p. T904 M. The mutation has a global allele frequency of 9.00 × 10^4^ and in the South Asian population (allele frequency = 0.00508961). This is a homozygous variant and has a recessive inheritance model.

### Family No. 10

Three individuals were included (10 − 1, 10 − 2, and 10 − 3) in the WGS screening. A total of 2580, 2349, and 3117 variants were obtained, respectively.

#### Detection of nonsynonymous SNV in the FOS, MIR452, PDHA1,and SERPINB1

A nonsynonymous variant, chr14:75281140 (C > T), was investigated in the Fos proto-oncogene, AP-1 transcription factor subunit *(FOS*) gene. This gene is located on chromosome 14q24.3. This variant occurs in exonic part of the gene that causes various nonsynonymous SNVs in different *FOS* isoforms including, FENST00000555686.1:exon3:c.C517T: p.R173C, ENST00000303562.9:exon4:c.C859T: p.R287C, ENST00000555347.1:exon2:c.C415T: p.R139C, and ENST00000535987.5:exon3:c.C751T: p.R251C, each involve in the substitution of arginine with cysteine, which affect the function of proteins. The mode of inheritance of this variant is autosomal recessive, and the patient is homozygous for this pathogenic variant.

The microRNA gene *MIR452* (MI0001733) is located on chromosome X at position (151959628–151959712). A homozygous nonsynonymous variant 151,959,925 T > C was identified in the *MIR452* gene. The transcript of this variant is ENST00000370328.4. This variant is located within both exonic and upstream regions and leads to an amino acid change in the *GABRE* gene, specifically in exon 6, where glutamic acid is replaced by glycine at position 233 (p.E233G). This homozygous variant follows an X-linked inheritance pattern; however, very low testicular expression is reported. Furthermore, no direct role in male infertility has been documented yet.

The next variant was detected *in the pyruvate dehydrogenase E1 subunit alpha 1* (*PDHA1*) gene with a total of 12 exons, located on chromosome Xp22.12 (Genomic coordinates (GRCh38): X:19,343,927 − 19,361,718). A nonsynonymous variant, chrX:19,359,639 A > C, was detected in the *PDHA1* gene, resulting in the substitution of several potential amino acids. This variant is existent in different isoforms of *PDHA1*, with variations at the following positions: exon 11 (c.A1159C, p.K387Q), exon 12 (c.A1273C, p.K425Q), exon 4 (c.A316C, p.K106Q), exon 11 (c.A1180C, p.K394Q), and exon 10 (c.A1066C, p.K356Q). This variant is X-linked, with the homozygous mode of inheritance; it may significantly impact enzyme activity, and potentially contribute to *PDHA1*-related disorder, such as pyruvate dehydrogenase deficiency.

The current study reported a nonsynonymous SNV (chr6:2833768 T > C) in the *Leukocyte Elastase Inhibitor (LEI*) *SERPINB1* gene, located on chromosome 6 (6p25.2, Genomic coordinates: 6:2,832,332–2,841,863) at position 2,833,768 with a total of 7 exons. This mutation leading to an amino acid change from asparagine to serine at codon 327 (p.N327S) in exon 7 of transcript ENST00000380739.6. This variant is in a homozygous state and follows a recessive mode of inheritance. *SER-PINB1* regulates inflammation and immune responses. This mutation may have functional impacts that warrant further study for a potential role in disease phenotypes.

### Family no. 11

WGS was performed for family 11, comprising 2 affected and 2 healthy individuals. Four members of family 11 were included (11 − 1, 11 − 2, 11 − 3, and 11 − 4) for performing NGS. 2535, 2356, 2645, and 2975 variants were obtained, respectively. Candidate variants were identified as segregating a within the family, protein-altering, affecting evolutionarily conserved residues, and predicted to be deleterious.

#### Detection of Novel frameshift deletion SNV in PRDM9, and SNV in KIAA1210

The *PRDM9* gene is located on chromosome 5 (5p14.2, Genomic coordinates (GRCh38): 5:23,507,155 − 23,528,093) with a total of 11 exons. WGS analysis revealed a frameshift deletion at position chr5:23527128_CACTCAC>−. In this frameshift deletion, the “ C A C T C A C” sequence is deleted (chr5:23527128_ CACTCAC>−). This variation occurs at exon 11. The transcript is ENST00000296682.4 and involves a deletion of nucleotides c.2040 to c.2046 in the coding sequence, causing protein alteration p.T681Rfs*256. The CADD score of 26.7 shows a strong pathogenic effect. This variant is likely to result in a truncated protein and may cause the loss of function of *PRDM9*.

Our study detected a nonsynonymous SNV in the *KIAA1210* gene, located on the chromosome X (Xq24, Genomic coordinates (GRCh38): X:119,078,635 − 119,151,130). This mutation is characterized by a position 119,116,637 A > G. This variant occurs at exon 4. The transcript is ENST00000402510.2. The variant causes an alteration c.T509C where leucine is replaced by proline at position 170. The variant is X-linked homozygous.

### Family no. 13

WGS was applied for family 13, comprising 4 affected and one healthy individual. 2156, 2145.

2161, 2035, and 3429 variants were obtained, respectively. Further review of these candidates for gene function and expression pattern led to the shortlisting of the following candidates with various clinical outcomes.

#### Investigation of nonsynonymous SNV in SPATA20, and G6PD

The *SPATA20* is involved in spermatogenesis and reproductive function. Our study investigated a nonsynonymous SNV in the Spermatogenesis Associated 20 (*SPATA20)* gene, located on chromosome 17 (17q21.33, Genomic coordinates (GRCh38): 17:50,547,174 − 50,555,852) at position 50,551,552, with a total of 6 exons. The transcript is ENST00000297213.8. In this variant, guanine (G) is replaced by adenine (A) (G > A). This mutation is homozygous recessive and occurs in the exonic region, causes an amino acid alteration, and leads to a truncated protein.

In this analysis, we found a nonsynonymous variant (SNV) in the *Glucose-6-phosphate dehydrogenase* (*G6PD)* gene, located on chromosome X (Xq28, Genomic coordinates (GRCh38): X:154,531,390 − 154,547,569) at position 154,534,362 A > G. It contains 13 exons. This exonic variant may potentially impact the function of the glucose-6-phosphate dehydrogenase (*G6PD*) enzyme. This variant is hemizygous and follows an X-linked inheritance pattern.

### Results family 15

NGS was performed for family 15, comprising 3 affected and 1 healthy individual. These members included 15 − 1, 15 − 2, 15 − 3, and 15 − 4 for performing NGS. 2532, 2534, 2488, and 431 variants were obtained, respectively.

#### Detection of the nonsynonymous variant in the SLC12A4

We identified a nonsynonymous SNV in the *SLC12A4* gene, located on chromosome 16 (16q22.1, Genomic coordinates (GRCh38): 16:67,943,474 − 67,968,694) at position 67,946,459, characterized by G > A. This gene comprises 24 exons. This mutation leads to an amino acid alteration that may impact the function of the K-Cl cotransporter encoded by *SLC12A4*. This is a homozygous recessive variant. This variant could have pathogenic relevance, particularly in disorders related to ion transport or cellular osmoregulation.

## Discussion

In the current study, we employed WGS to investigate genetic variants that are responsible for familial male infertility. By using comprehensive variant annotation, stringent bioinformatic tools, several novel, rare hemizygous and homozygous SNVs are detected. Several of the identified variants have high CADD scores ≥ 20, indicating that these changes may potentially affect the protein structure and function. CAVIN 2 may play a pivotal role in testosterone biosynthesis by activating protein kinase C PKC by Ca2 + influx into the cytoplasm [22]. In spermatogenesis and hormone production, Ca and intra-cellular Ca 2 + binding proteins are involved in the rat testis. Calbindin D-28 K and the calmodulin superfamily were localized in the Leydig cells, which are sites of testosterone synthesis. At all stages of development, in fetal-type Leydig cells and adult-type Leydig cells, Calbindin D-28 K-immunoreactivity was recorded. In developing spermatocytes and maturing spermatids, the presence of Calmodulin is evidenced by Calmodulin staining. The localization and appearance of these proteins point towards their role in Leydig cell function and spermatogenesis [23]. CALB 2 Knock-out mice show vulnerable fertility [22]. In humans, testicular cell biopsies CALB 2 is detected in immature Sertoli cells, which shows its involvement in spermatogenesis [24]. In a study where testis tissue samples were taken for localization of c-fos, phosphorylated c-fos. Immunohistochemistry showed its expression and localization in spermatogonia, spermatocytes, and spermatids, and in Sertoli cells [25].

The microRNA gene miR-452, like other microRNAs, may have a regulatory function with various genes responsible for spermatogenesis. A defective miRNA-mRNA complex leads to defective maturation and production of sperm, eventually leading to infertility [26]. A study designed to assess gene expression of miRNA in infertile males shows significantly high expression for miRNA-122 in azoospermia patients and oligospermia patients. In the mouse model, double knock-out of miR-34b/c and miR-449 results in severe disorder in spermatogenesis. Another study reveals disturbed spermatozoa maturation resulting in asthenospermia due to deletion of miR34b/c and miR-449 in mice (Papaioannou and Nef 2010). miRNA-191–5p is associated with sperm differentiation.

Our study has revealed several rare hemizygous variants in X-linked genes, including *PDHA1* (19359639 A > C, *G6PD* (154534362 A > G) All these X-linked variants play a pivotal role in reproduction and fertility.

*TJP2* stands for “Tight junction protein” and encodes zonula occludens, which is a member of the membrane–associated guanylate kinase homolog family. TJP2 is an integral protein bound to the actin cytoskeleton by adaptor proteins. Most prominent of which are claudins and occludins, mainly zonula occludens proteins ZO-1, ZO-2, and ZO-3, which are associated with the cytoplasmic surface of the tight junctions and organize a complex. Loss of tight junction integrity in Sertoli cells could disrupt the BTB, disrupting spermatogenesis, which may lead to infertility. These proteins are localized to the blood-testis barrier region, so ZO-2 plays a key role in blood-testis barrier integrity and male fertility [27]. Tight junctions between Sertoli cells make up the major component of the blood-testis barrier in the seminiferous epithelium. In CIS disturbed distribution of these junction proteins ZO-1 and ZO-2 in Sertoli cells reveals loss of function in the blood-testis barrier [28].

Drug interaction study on male mice reveals regulatory role of *SLC12A4* and *Mt-nd6* in sperm mitochondria along with *Catsperb*, *Greb1*, *Mospd2*, *Esrrb*, *Specc1*, *and Sohlh1* linkage to estrogen response and sperm penetration function, so it can be concluded that some drugs alter the expression of mRNA in the reproductive system [29]. The RT-nested PCR indicates the presence of KCC3 SLC12A6, and KCC4 SLC12A7 in the mRNA of sperm, which strongly supports the role of these transporter/carrier proteins in RVD, thus aiding in the penetration of sperm into the egg [30].Furthermore, SNVs were also detected in genes such as*SERPINB1*. To summarize, WGS effectively identified novel and potentially pathogenic variants that are linked with male infertility in Pakistani families. Furthermore, the recurrence of shared variants across multiple families and their implication in cellular and reproductive processes support potential etiologies in idiopathic male infertility.

Despite the use of advanced NGS technology for discovering new and novel variants in the study population, several limitations were also noted. Limitations of the study includes a smaller sample size and a lack of functional validation of the detected variants. That makes it difficult to make a decisive conclusion about the genotype-phenotype correlations. Our analysis disclosed a set of rare and novel variants in both autosomal and X-linked genes. The findings further improve our understanding of the pathophysiology of male infertility and underscore novel gene-disease associations. Replication of the same experimental model on larger cohorts in vitro assays followed by validation through animal models, is required to elucidate the pivotal role of the identified variants and to establish their clinical significance.

## Supplementary Material

Supplementary Table 1 Supplementary Material 1: Table 1. Filtered variants for family 1.

Supplementary Table 2 Supplementary Material 2: Table 2. Filtered variants for family 10.

Supplementary Table 3 Supplementary Material 3: Table 3. Filtered variants for family 11.

Supplementary Table 4 Supplementary Material 4: Table 4. Filtered variants for family 13.

Supplementary Table 5 Supplementary Material 5: Table 6. Filtered variants for family 14.

The online version contains supplementary material available at https://doi.org/10.1186/s43042-025-00828-y.

## Figures and Tables

**Fig. 1:**
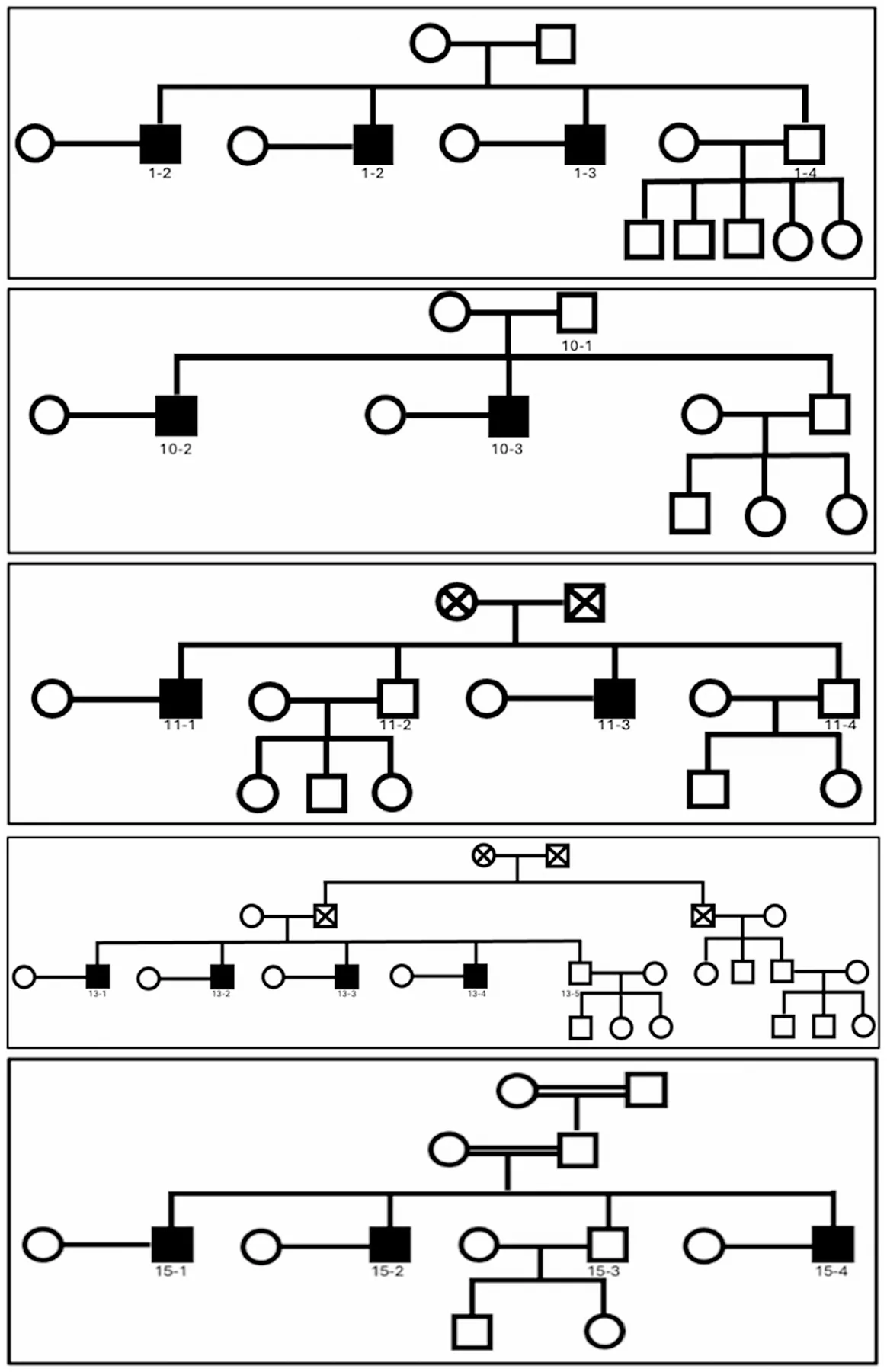
Pedigree diagram of family (1, 10, 11, 13, and 15) showing segregation pattern supports an autosomal recessive (or X-linked, as applicable) mode of inheritance of the detected variant. Squares represent males and circles represent females. Filled symbols indicate affected individuals, while open symbols denote unaffected members. A diagonal line through a symbol represents a deceased individual

**Fig. 2:**
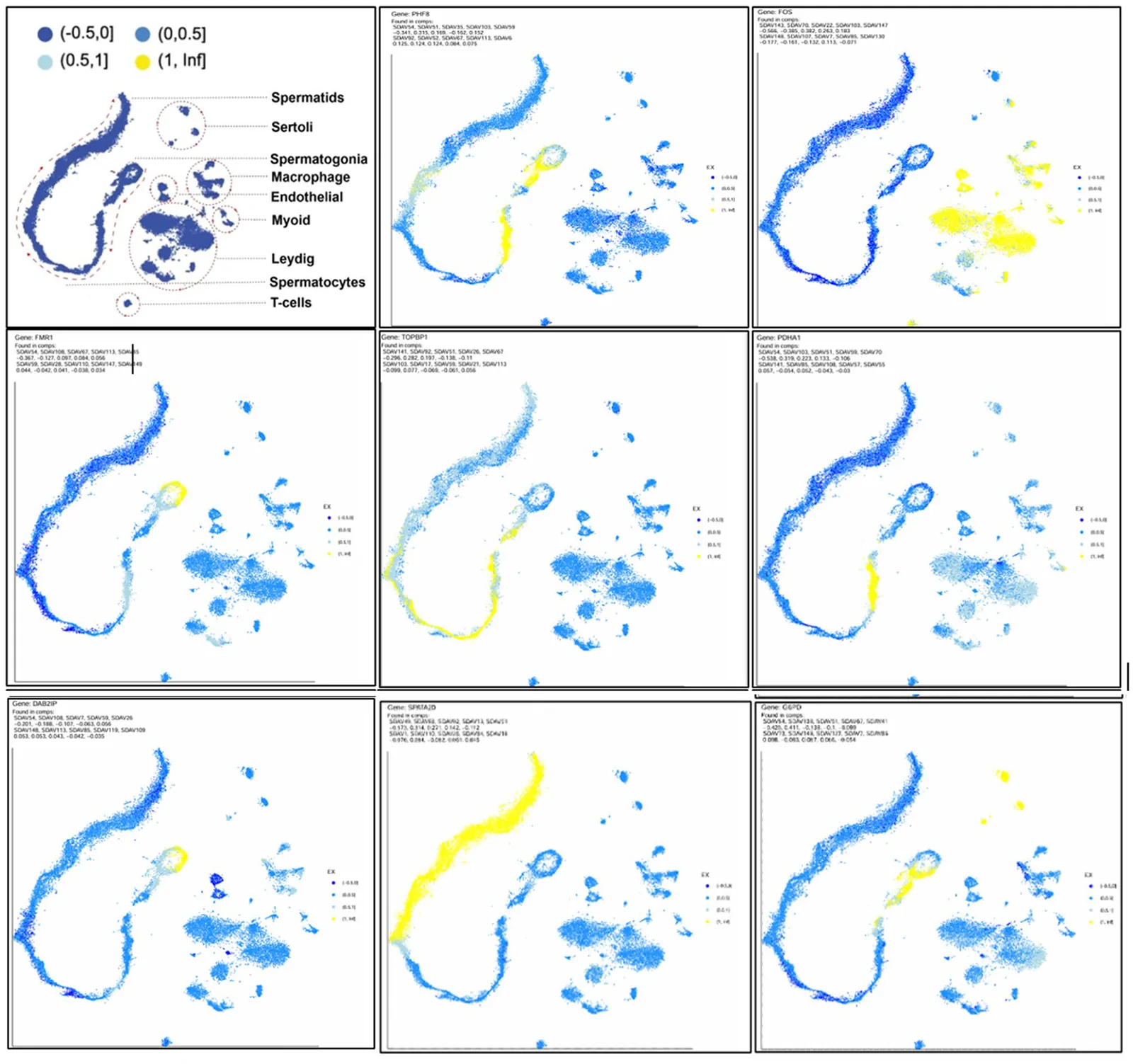
The expression profiles of the candidate genes (*PRDM9*, *KIAA1210*, *SPATA20*, *G6PD*, *TJP2*, *PRSS51*, *SLC12A4*) are shown. Single-cell resolution within cells of the adult testis, as summarized by the Human Infertility Single-Cell Testis Atlas (HISTA)

**Fig. 3:**
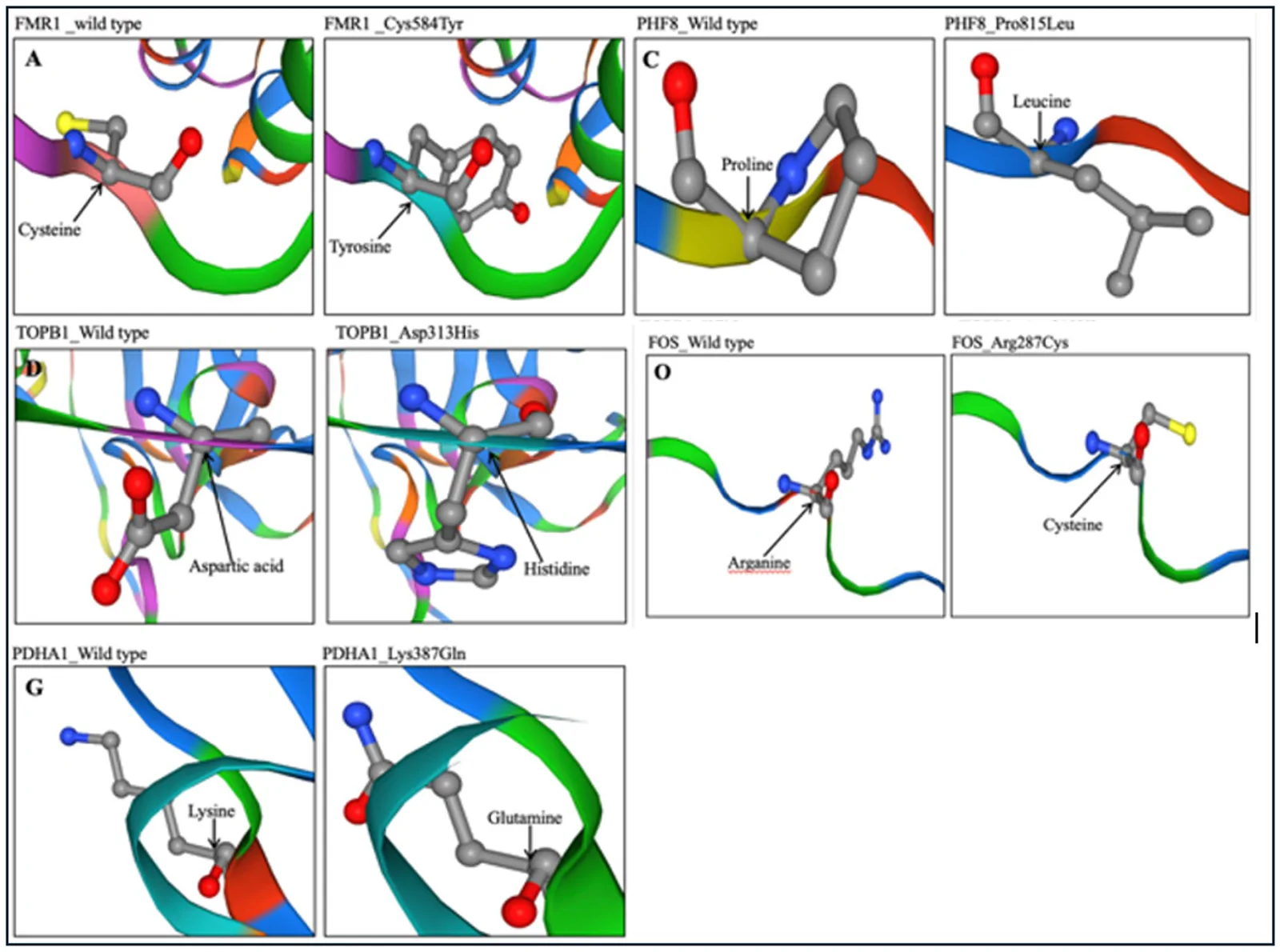
Illustrated 3D models of the representative variants. The wild type and mutated amino acids are denoted with an arrow

**Table 1:** A spectrum of sperm morphological abnormalities was observed in the cases

Family ID	Patient ID	Semen volume (ml)	Normal semen volume (mL)	Sperm count million/ejaculate	Normal sperm count (million/mL)	Motility	Remarks
Family No.1	1–1	2	1.4	00	16	00	Azoospermic
	1–2	2	1.4	00	16	00	Azoospermic
	1–3	2.5	1.4	00	16	00	Azoospermic
	1–4	Normal		Normal	16	Normal	Normal
	10–1	Normal		Normal	16	Normal	Normal
Family No.10	10–2	2.0	1.4	10	16	55	Oligospermic
	10–3	2.5	1.4	65	16	15	Asthenospermic
	11–1	2.5	1.4	00	16	00	Azoospermic
Family No.11	11–2	Normal		Normal	16	Normal	Normal
	11–3	2.0	1.4	00	16	00	Azoospermic
	11–4	Normal		Normal	16	Normal	Normal
	13–1	1.0	1.4	00	16	00	Azoospermic
	13–2	0.5	1.4	65	16	30	Asthenospermic
Family No.13	13–3	2.0	1.4	35	16	25	Asthenospermic
	13–4	2	1.4	35	16	26	Asthenospermic
	13–5	Normal		Normal	16	Normal	Normal
	15–1	3.0	1.4	00	16	00	Azoospermic
Family No.15	15–2	2.5	1.4	05	16	10	Asthenooligospermic
	15–3	Normal		Normal	16	Normal	Normal
	15–4	2.0	1.4	10	16	10	Asthenooligospermic

The reference value is according to the latest (6th) edition of the WHO laboratory manual for the examination and processing of human semen (2021), the lower reference limits are based on the 5th percentile values

**Table 2:** Detected variants with family ID, type of variant, transcript details, CADD score, Rs No, and disease model, ACMG classification for variant pathogenicity

Gene name	ID	Key	AAChange.wgEncodeGencodeBasicV35	CADD score	Dz.Model	gnomadExomes.v2.1.1_rsId	ACMG classification
FMR1	1–4	chrX:147948696_G > A	FMR1:ENST00000218200.12:exon16:c.G1688A: p.C563Y, FMR1:ENST00000370475.9:exon17:c.G1751A: p.C584Y, FMR1:ENST00000370471.7:exon16:c.G1480A: p.A494T, FMR1:ENST00000440235.6:exon16:c.G1613A: p.C538Y	22.7	X-linked-hem	rs587780338	VUS
FMR1	1–1	chrX:147948696_G > A	FMR1:ENST00000218200.12:exon16:c.G1688A: p.C563Y, FMR1:ENST00000370475.9:exon17:c.G1751A: p.C584Y, FMR1:ENST00000370471.7:exon16:c.G1480A: p.A494T, FMR1:ENST00000440235.6:exon16:c.G1613A: p.C538Y	22.7	X-linked-hem	rs587780338	VUS
PHF8	1–1	chrX:53985021_G > A	PHF8:ENST00000338946.10:exon17:c.C2033T: p.P678L, PHF8:ENST00000357988.9:exon18:c.C2444T: p.P815L, PHF8:ENST00000338154.11:exon18:c.C2336T: p.P779L, PHF8:ENST00000322659.12:exon19:c.C2285T: p.P762L	20.8	X-linked-hem	rs1557099079	VUS
PHF8	1–2	chrX:53985021_G > A	PHF8:ENST00000338946.10:exon17:c.C2033T: p.P678L, PHF8:ENST00000357988.9:exon18:c.C2444T: p.P815L, PHF8:ENST00000338154.11:exon18:c.C2336T: p.P779L, PHF8:ENST00000322659.12:exon19:c.C2285T: p.P762L	20.8	X-linked-hem	rs1557099079	VUS
PHF8	1–4	chrX:53985021_G > A	PHF8:ENST00000338946.10:exon17:c.C2033T: p.P678L, PHF8:ENST00000357988.9:exon18:c.C2444T: p.P815L, PHF8:ENST00000338154.11:exon18:c.C2336T: p.P779L, PHF8:ENST00000322659.12:exon19:c.C2285T: p.P762L	20.8	X-linked-hem	rs1557099079	VUS
TOPBP1	1–2	chr3:133652615_C > G	TOPBP1:ENST00000260810.10:exon8:c.G937C: p.D313H	31	REC-hom	rs1353662227	VUS
DAB2IP	1–1	chr9:121773239_C > T	DAB2IP: ENST00000309989.1:exon10:c.C2339T: p.T780M, DAB2IP: ENST00000259371.6:exon12:c.C2627T: p.T876M, DAB2IP: ENST00000648444.1:exon10:c. C2528T: p.T843M, DAB2IP: ENST00000408936.7:exon12:c.C2711T: p.T904M	21.2	REC-hom	rs183735294	VUS
DAB2IP	1–3	chr9:121773239_C > T	DAB2IP: ENST00000309989.1:exon10:c. C2339T: p.T780M, DAB2IP: ENST00000259371.6:exon12:c.C2627T: p.T876M, DAB2IP: ENST00000648444.1:exon10:c. C2528T: p.T843M, DAB2IP: ENST00000408936.7:exon12:c.C2711T: p.T904M	21.2	REC-hom	rs183735294	VUS
DAB2IP	1–4	chr9:121773239_C > T	DAB2IP: ENST00000309989.1:exon10:c.C2339T: p.T780M, DAB2IP: ENST00000259371.6:exon12:c.C2627T: p.T876M, DAB2IP: ENST00000648444.1:exon10:c.C2528T: p.T843M, DAB2IP: ENST00000408936.7:exon12:c.C2711T: p.T904M	21.2	REC-hom	rs183735294	VUS
DAB2IP	1–2	chr9:121773239_C > T	DAB2IP: ENST00000309989.1:exon10:c.C2339T: p.T780M, DAB2IP: ENST00000259371.6:exon12:c.C2627T: p.T876M, DAB2IP: ENST00000648444.1:exon10:c.C2528T: p.T843M, DAB2IP: ENST00000408936.7:exon12:c.C2711T: p.T904M	21.2	REC-hom	rs183735294	VUS
FOS	10–3	chr14:75281140_C > T	FOS: ENST00000555686.1:exon3:c.C517T: p.R173C, FOS: ENST00000303562.9:exon4:c.C859T: p.R287C, FOS: ENST00000555347.1:exon2:c.C415T: p.R139C, FOS: ENST00000535987.5:exon3:c.C751T: p.R251C	26.6	REC-hom	rs146332374	VUS
MIR452	10–3	chrX:151959925_T > C	GABRE: ENST00000370328.4:exon6:c.A698G: p.E233G	23.8	X-linked-hem	rs370193189	VUS
PDHA1	10–1	chrX:19359639_A > C	PDHA1:ENST00000422285.7:exon11:c.A1159C: p.K387Q, PDHA1:ENST00000379806.9:exon12:c.A1273C: p.K425Q, PDHA1:ENST00000379804.1:exon4:c. A316C: p.K106Q, PDHA1:ENST00000545074.5: exon11:c.A1180C: p. K394Q, PDHA1:ENST00000540249.5:exon10:c.A1066C: p.K356Q	22.9	X-linked-hem	rs201156613	Conflicting_interpretations_of_pathogenicity
SERPINB1	10–3	chr6:2833768_T > C	SERPINB1:ENST00000380739.6:exon7:c.A980G: p.N327S	24.6	REC-hom	rs754581913	VUS
PRDM9	1–2	chr5:23527128_CACTCAC>-	PRDM9:ENST00000296682.4:exon11:c.2040_2046del: p.T681Rfs*256	26.7	REC-hom	No RS number	Likely pathogenic
KIAA1210	11–1	chrX:119116637_A > G	KIAA1210:ENST00000402510.2:exon4:c. T509C: p.L170P	23.1	X-linked-hem	rs746297253	VUS
SPATA20	13–5	chr17:50551552_G > A	SPATA20:ENST00000634597.1:exon12:c.G1570A: p.G524S, SPATA20:ENST00000619622.4:exon12:c.G1438A: p.G480S, SPATA20:ENST00000356488.8:exon12:c.G1570A: p.G524S, SPATA20:ENST00000006658.11:exon13:c.G1618A: p.G540S	23.2	REC-hom	rs746297253	VUS
SPATA20	13–4	chr17:50551552_G > A	SPATA20:ENST00000634597.1:exon12:c. G1570A: p.G524S, SPATA20:ENST00000619622.4:exon12:c.G1438A: p.G480S, SPATA20:ENST00000356488.8:exon12:c.G1570A: p.G524S, SPATA20:ENST00000006658.11:exon13:c.G1618A: p.G540S	23.2	REC-hom	rs746297253	VUS
G6PD	13–5	chrX:154534362_A > G	G6PD: ENST00000393562.10:exon6:c.T620C: p.M207T, G6PD: ENST00000393564.6:exon6:c.T620C: p.M207T, G6PD: ENST00000369620.6:exon6:c.T620C: p.M207T	25.7	X-linked-hem	rs398123879	VUS
SLC12A4	15–2	chr16:67946459_G > A	SLC12A4:ENST00000572037.5:exon17:c.C2272T: p.R758C, SLC12A4:ENST00000422611.6:exon17:c.C2422T: p.R808C, SLC12A4:ENST00000316341.8:exon18:c.C2416T: p.R806C, SLC12A4:ENST00000576616.5:exon18:c.C2416T: p.R806C, SLC12A4:ENST00000537830.6:exon18:c.C2398T: p.R800C, SLC12A4:ENST00000541864.6:exon18:c.C2323T: p.R775C	23.9	REC-hom	rs372127326	VUS

## Data Availability

The data that support the findings of this study are available as electronic supplementary files.
